# Genetic mutational analysis of pediatric acute lymphoblastic leukemia from a single center in China using exon sequencing

**DOI:** 10.1186/s12885-020-6709-7

**Published:** 2020-03-12

**Authors:** Honghong Zhang, Hongsheng Wang, Xiaowen Qian, Shuai Gao, Jieqi Xia, Junwen Liu, Yanqin Cheng, Jie Man, Xiaowen Zhai

**Affiliations:** 1 Department of Hematology oncology, Children’s hospital of Fudan university, 399 Wanyuan Road, Shanghai, China; 2 Clinical laboratory center, Children’s hospital of Fudan University, Shanghai, China

**Keywords:** Acute lymphoblastic leukemia, Genomics, Molecular pathogenesis, Pediatrics, KMT2D

## Abstract

**Background:**

Acute lymphoblastic leukemia (ALL), the most common childhood malignancy, is characterized by recurring structural chromosomal alterations and genetic alterations, whose detection is critical in diagnosis, risk stratification and prognostication. However, the genetic mechanisms that give rise to ALL remain poorly understood.

**Methods:**

Using next-generation sequencing (NGS) in matched germline and tumor samples from 140 pediatric Chinese patients with ALL, we landscaped the gene mutations and estimated the mutation frequencies in this disease.

**Results:**

Our results showed that the top driver oncogenes having a mutation prevalence over 5% in childhood ALL included *KRAS* (8.76%), *NRAS* (6.4%), *FLT3* (5.7%) and *KMT2D* (5.0%). While the most frequently mutated genes were *KRAS*, *NRAS* and *FLT3* in B cell ALL (B-ALL), the most common mutations were enriched in *NOTCH1* (23.1%), *FBXW7* (23.1%) and *PHF6* (11.5%) in T cell ALL (T-ALL). These mutant genes are involved in key molecular processes, including the *Ras* pathway, the *Notch* pathway, epigenetic modification, and cell-cycle regulation. Strikingly, more than 50% of mutations occurred in the high-hyperdiploid (HeH) ALL existed in *Ras* pathway, especially *FLT3* (20%). We also found that the epigenetic regulator gene *KMT2D*, which is frequently mutated in ALL, may be involved in driving leukemia transformation, as evidenced by an in vitro functional assay.

**Conclusion:**

Overall, this study provides further insights into the genetic basis of ALL and shows that Ras mutations are predominant in childhood ALL, especially in the high-hyperdiploid subtype in our research.

## Background

Acute lymphoblastic leukemia (ALL), the most common childhood tumor, results from the clonal proliferation of lymphoid stem or progenitor cells with arrested maturation, with more than 80% originating from B cell progenitors [1]. ALL is characterized by recurring structural chromosomal alterations, including aneuploidy (high-hyperdiploid, chromosomes ≥51; hypodiploid, chromosomes ≤44) and translocations (e.g., t (12;21)/*ETV6-RUNX1*, t (1;19)/*TCF3-PBX1*, t (9;22)/*BCR-ABL1*, and *KMT2A* (also known as *MLL*) rearrangement). However, chromosomal changes alone are often insufficient to trigger leukemia, some additional genetic alterations must contribute to tumorigenesis [2, 3].

Cytogenetic alterations or molecular abnormalities are frequent, and several molecular markers have been identified to stratify risk and predict prognosis, as they play key roles in ALL pathogenesis. Specific ALL subtypes exhibit different mutation distributions; for example, *TP53* mutations mostly occur in hypodiploidy [4, 5]. *PAX5/IKZF1* copy number abnormalities frequently exist in B-ALL, whereas mutations within *NOTCH1*, *FBXW7,* and *CDKN2A/CDKN2B* are enriched in T-ALL [1, 6–8]. Rare germline mutations in the genes *PAX5* [9] and *ETV6* [10] were found to be linked to familial leukemia, and some chemical agents or radiation exposure could increase the incidence of leukemia [6]. In addition, some molecular alterations, such as *CREBBP* [11–13], *NT5C2* [14, 15] and *PRPS1* mutations [16], are associated with chemo-resistance. Thus, the identification of these abnormalities not only reveals molecular pathology, but also provides important therapeutic targets. Some targetable alterations or pathways have been used for therapeutic interventions in the clinic, especially kinase-activating alterations in *BCR-ABL1*-positive or Philadelphia chromosome-like ALL patients who are amenable to tyrosine kinase inhibitors with improved survival rates [17, 18]. However, a substantial percentage of patients classified as having a “good prognosis” (e.g., t (12;21)/*ETV6-RUNX1* or high-hyperdiploid) still experience relapse, which may be caused by the existence of additional or secondary molecular variants. Therefore, it remains important to further identify the repertoires of gene mutations and understand its clinical significance in pediatric ALL.

Recently, genetic profiling of several subtypes of pediatric ALL has been conducted with NGS [4, 5, 11, 19, 20]. Numerous germline genetic variants and somatic alterations have been identified in newly diagnosed and relapsed childhood ALL or in specific subtypes, which may also have prognostic implications [19, 20]. NGS has revealed changes in the microarchitecture and gene sequence, which advanced the understanding of the molecular basis of ALL and complemented genetic features of the ALL subtypes.

In this study, we used targeted exome sequencing technology to reveal the mutational spectrum in patients with ALL at initial diagnosis to better understand the cytogenetic and molecular classification of pediatric ALL in Chinese children, which may lead to the discovery of new therapeutic targets and enable the development of a tailored therapeutic regimen for each patient.

## Methods

### Sample collection and genomic DNA extraction

A total of 140 pediatric patients (≤18 years) with ALL enrolled consecutively in this study were newly diagnosed and treated in the children’s hospital of Fudan University in China between January 2015 and December 2017. ALL diagnosis was established by analysis of leukemic cells with morphology, immunophenotyped, and cytogenetics. Immunophenotype (B-ALL or T-ALL) was defined according to the European Group for the Immunological Characterization of Leukemias. Informed consent was obtained in accordance with the Declaration of Helsinki and approved by the Institutional Review Board of the Fudan Institutes. Bone marrow samples were collected at initial diagnosis; matched remission samples or fingernails were used as germline controls. Genomic DNA was extracted from cell pellets using DNAeasy Blood and Tissue Kit (Qiagen, USA). DNA was quantified using a Qubit Fluorometer (Life Technologies, USA), and DNA integrity was assessed by agarose gel electrophoresis. The transcripts of *BCR-ABL1*, *ETV6-RUNX1*, *TCF3-PBX1*, *SIL-TAL1* fusion genes, and *MLL* rearrangement (*MLLr*) were detected with reverse transcriptase polymerase chain reaction (RT-PCR) or fluorescence in situ hybridization (FISH) as previously described [21].

### Targeted capture sequencing and mutation analysis

Targeted capture libraries were prepared, and the exons of 950 genes related to cancer were selected for sequencing (Table S1). Genomic DNA samples were sheared by sonication, and the sheared genomic DNA was then hybridized with a NimbleGen 2.0 probe sequence capture array of Roche (http://www.nimblegen.com/products/seqcap/ez/v2/index.html) to enrich the exonic DNA (Joy Orient, China). The libraries were first tested for enrichment by qPCR and for size distribution and concentration using the Agilent Bioanalyzer 2100. The samples were then sequenced on an Illumina Hiseq2500, and two parallel reactions were performed for each sample. Raw image files were processed by BclToFastq (Illumina) for base calling and generating the raw data. The low-quality variations were filtered out using a quality score ≥ 20 (Q20). The sequencing reads were aligned to the NCBI human reference genome (hg19) using BWA (version 0.5.10), including coverage and quality assessment, single-nucleotide variant (SNV) and indel detection, annotation and prediction of deleterious effects for sequence mutations. Samtools and Pindel were used to analyze single-nucleotide polymorphisms (SNPs) and indels in the sequence. Synonymous changes and SNPs with MAF (minor allele frequency) higher than 5% were removed (http://www.ncbi.nlm.nih.gov/projects/SNP). Nonsynonymous changes and small indels were filtered using SIFT (version 1.03), PolyPhen-2 (version 2.2.2), PROVEAN (version 1.1.3), and MutationTaster 2. All candidate mutations were filtered with minimum coverage ≥10, minimum tumor variant frequency ≥ 0.10, normal variant frequency ≤ 0.05, and candidate driver mutations were considered as two prediction algorithms to be significant or identified as recurrent in COSMIC.

### Generation of KMT2D knockdown cell lines

Lentivirus-mediated gene-specific small hairpin RNAs (shRNAs) were used to knockdown the expression of the *KMT2D* gene in Nalm-6 cells (human ALL pre-B cells). Nalm-6 cells were obtained from FuDan IBS Cell Center (FDCC) and tested for mycoplasma (catalog no. FDCC-HGN101) and were cultured in RPMI1640 (Gibco, USA) medium with 10% FBS (Gibco, USA) and 1% penicillin-streptomycin (Gibco, USA). shRNA-targeted sequences (Table S2) were subcloned into the lentiviral vector pLenO-GTP, and then plasmids and packaging vectors (pRsv-REV, pMDlg-pRRE, pMD2G, pLenO-GTP) were cotransfected into HEK293T cells to generate lentivirus. These vectors were obtained from BioLink Laboratory (Shanghai, China). A total of 5 × 10^4^ cells/μl were infected with MOI = 100 IU/ml virus and 5 μg/ml of polybrene (Sigma-Aldrich, Germany) by spin-down infection at 1400 rpm for 2 h; 1 μg/ml puromycin (Sigma-Aldrich, Germany) was used to select stable cell lines 3 days later. Three biologically independent replicates were carried out. Reverse-transcription quantitative real-time PCR (RT-qPCR) was performed to measure the knockdown effect of shRNA.

Total RNA was extracted from infected cells using the RNeasy Mini Kit (Qiagen, USA), and 1 μg of RNA was reverse transcribed using the PrimeScript RT reagent Kit with gDNA Eraser (Takara, Japan) and qPCR amplifications using TB Green Premix Ex Taq II (Takara, Japan). GAPDH was used as a reference gene (Table S3). Cell proliferation was detected using a Cell Counting Kit-8 (CCK-8) (Dojindo, Japan) in 96 well plates according to the manufacturer’s instructions. The cell cycle (CycleTest PLUS DNA Reagent Kit, catalog no. 340242) and the cell apoptosis (Annexin V-PE Apoptosis Detection Kit, catalog no. 559763) analysis were measured by BD flow cytometry according to the manufacturer’s instructions.

### Statistical analysis

We used SPSS 24.0 (SPSS, Chicago) statistical software for statistical analysis. Comparisons of categorical variables were determined by Pearson’s chi-squared test or Fisher’s exact test. Two-sided *P* < 0.05 was considered statistically significant.

## Results

### Somatic mutations in newly diagnosed ALL patients

To better understand the landscape of somatic mutations in Chinese children with ALL, we performed targeted sequencing of 140 pediatric ALL patients (114 B-ALL and 26 T-ALL) with matched germline and diagnostic samples. The average sequencing coverage reached 634.06X (range 109.17X~ 1149.39X) and 128.43X (45.58X~ 500.37X), respectively, in tumor and control samples, which allowed accurate determination of mutant allele fractions and somatic mutation analysis (Figure S1A). In total, we detected 2193 somatic SNVs, 87 deletions and 56 insertions in the 950 sequenced genes. The average number of somatic mutations detected was 8.8 (range 0~78) per patient, including the nonsynonymous and synonymous mutations. We found no correlation between the number of mutations and gender, age, and initial white blood cell (WBC) counts. There was a trend towards more somatic mutations in T-ALL (average 8.0) than in B-ALL (average 6.0), although no significant difference (*P* = 0.267) was achieved plausibly because of limited sample size (Figure S1B). Basic characteristics of patients are described in Table S4.

Mutational spectrum analysis revealed that C > T single-base substitution was the dominant mutational event, which has been observed in all common cancer types and is likely caused by a spontaneous endogenous deamination process [22, 23]. By comparison, we found that B-ALL and T-ALL showed the highest rates of C > T (39.5%) and T > C (26%) substitutions, respectively (Figure S1C). The allele fractions (AFs) of SNVs were binomially distributed with a major peak around AF 0.15 (Figure S1D), suggesting a large fraction of somatic mutations were from the subclones.

### Mutational landscape of pediatric ALL

To identify the somatic mutations of potential pathogenicity in ALL, we excluded several genes whose protein sequences and structural changes were not predicted to be deleterious (materials and methods). Based on 261 (25.9%) of the non-silent mutations predicted to be deleterious, we estimated the mutation prevalence and found that recurrently mutated genes with a mutation prevalence over 5% included *KRAS* (8.76%), *NRAS* (6.4%), *FLT3* (5.7%) and *KMT2D* (5.0%) in childhood ALL. The most frequently mutated genes were members of the *Ras* signaling pathway (*KRAS*, *NRAS*, *FLT3*, *NF1*, *PTPN11*), especially in HeH, where 50% mutations occurred in the *Ras* pathway. In 30 HeH patients, *FLT3* had the most recurrent mutations with a mutation prevalence of 20, and 75% *FLT3* mutations occurred in HeH.

**Fig. 1:**
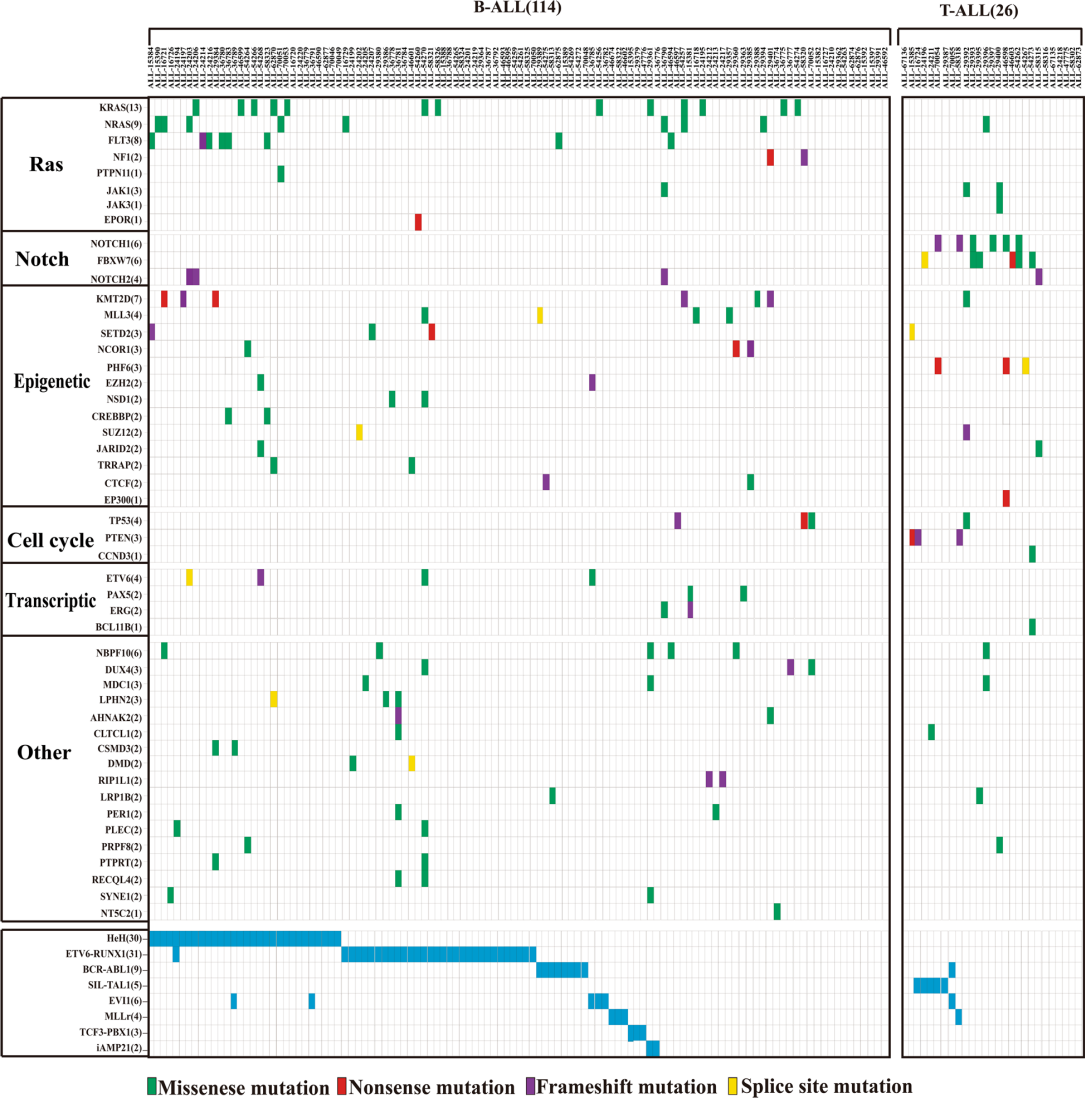
Mutational landscape of newly diagnosed 140 pediatric ALL patients. Heatmap diagram showing genomic data of 140 ALL patients, each of which is represented by a column, and each row represents a gene. Each color box indicates a type of mutation

We observed obvious differences in terms of mutational landscape between B-ALL and T-ALL patients. The most frequently mutated genes were *KRAS* (11.4%), *NRAS* (7.0%), *FLT3* (7.0%), and *KMT2D* (5.3%) in B-ALL, whereas *NOTCH1* (23.1%), *FBXW7* (23.1%), *PHF6* (11.5%) and *PTEN* (11.5%) were enriched in T-ALL. The most prevalent mutations were enriched in the *Ras* signaling pathway (*KRAS, NRAS, FLT3, NF1*) and *Notch* pathway (*NOTCH1, FBXW7*) in B-ALL and T-ALL, respectively (Fig. 1). We also found that somatic mutations in the *Ras* signaling pathway displayed a similar pattern in which few mutations coexisted in patients with recurrent translocations (i.e., *ETV6-RUNX1*, *BCR-ABL1*, *MLLr* and *TCF3-PBX1*). However, mutations in the *Notch* signaling pathway were often shown in patients with fusions of *SIL-TAL1* and *MLLr* (Table 1). When further calculating the number of pathogenetic genes within ALL subtypes, we found that two patients with intrachromosomal amplification of chromosome 21 (*iAMP21*) had a higher mutation burden (11.5/patient), however, the sample size is limited and the results need to be verified.

**Table 1:** Genetic subtypes and number of pathogenetic mutations in ALL patients (*n* = 140)

Subtype^a^	No. of patients	No. of mutations Per patients	Patients with Ras mutations^b^	Patients with Notch mutations^c^
*ETV6-RUNX1*	31	2.1 (0~20)	3 (9.7%)	0 (0%)
HeH	30	1.6 (0~5)	15 (50.0%)	0 (0%)
*BCR-ABL1*	9	0.8 (0~2)	1 (11.1%)	0 (0%)
*EVI1*	6	0.7 (0~1)	1 (16.7%)	0 (0%)
*SIL-TAL1*	5	2.8 (0~5)	0 (0%)	2 (40%)
*MLLr*	4	1.8 (0~5)	0 (0%)	1 (25%)
*TCF3-PBX1*	3	0	0 (0%)	0 (0%)
*iAMP21*	2	11.5 (1~21)	1 (50.0%)	0 (0%)
Hypodiploidy	2	1.0 (0~2)	0 (0%)	0 (0%)

^a^*HeH* High-hyperdiploid (51~67 chromosomes), *iAMP21* Intrachromosomal amplification of chromosome 21

^b^ Number of significant mutations in the genes *KRAS*, *NRAS*, *FLT3*, *NF1* and *PTPN11*

^c^ Number of significant mutations in the genes *NOTCH1* and *FBXW7*

### Recurrently targeted pathways in pediatric ALL

#### Mutations in the Ras signaling pathway were more abundant in B-ALL

**Fig. 2:**
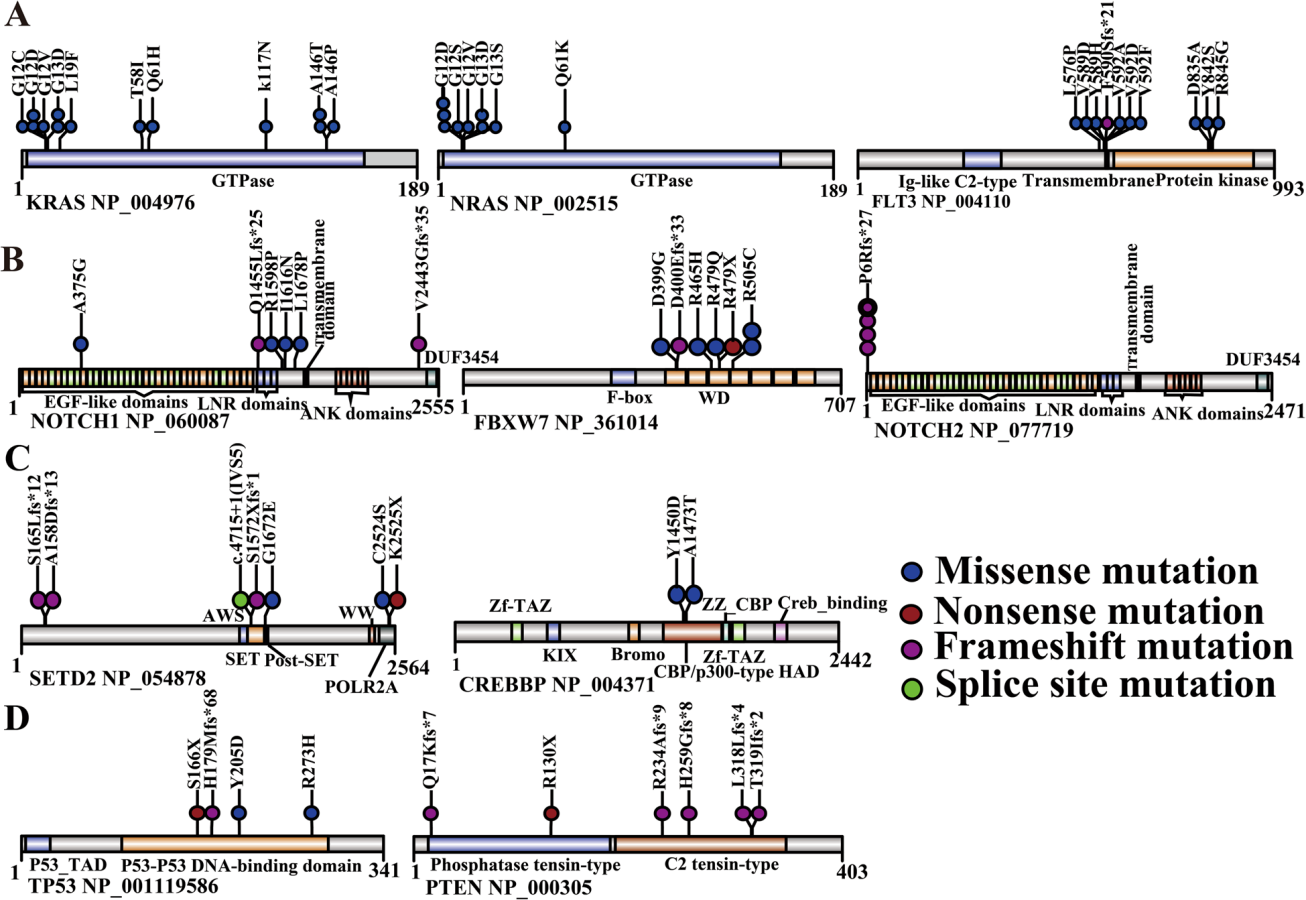
Recurrent somatic mutations in diagnostic ALL patients Schematic of protein structures showing mutations recurrently identified in diagnostic ALL samples. Proteins involved in the *Ras* pathway (**a**), *Notch* pathway (**b**), Epigenetic regulators (**c**) and cell cycle (**d**).

The most frequently mutated genes were members of the *Ras* signaling pathway, and Ras mutations were more abundant in B-ALL. The well-known hotspot mutations in the *Ras* genes included G12C/D/S/V (*KRAS* = 4; *NRAS* = 5), G13D/S/V (*KRAS* = 2; *NRAS* = 3), Q61K/H (*KRAS* = 1; *NRAS* = 1) and other mutational sites, such as A146T/P (*KRAS* = 3) and K117N (*KRAS* = 1). Interestingly, we found that one patient harbored both *KRAS* (G12C) and *NRAS* (G12D) mutations simultaneously with AFs less than 0.2, implying that at least two leukemia clones existed (Figs. 1 and 2a), however the patient with primary bone marrow blasts more than 97%.

*FLT3* plays a key role in hematopoietic cell growth and survival, which codes for a cell surface tyrosine kinase receptor. It was the most frequently altered gene in HeH in our research, and somatic mutations in *FLT3* predominantly occurred in the tyrosine kinase domain and juxtamembrane domain, with the D835 residue as the most frequently mutated site [24]. Here, we identified several novel recurrent mutational sites in the kinase domain (D835A, Y842S, R845G) and in the transmembrane region (V592A, V592D, V592F), which may be involved in the regulation of *FLT3* dimerization and self-activation. No FLT3-ITD mutations were detected in the entire cohort (Fig. 2a).

In addition, loss-of-function mutations in the *Ras* signaling negative regulator (*NF1)* occurred in 2 patients: R1306X (nonsense mutation) and R652 Vfs*36 (frameshift mutation). *PTPN11* encodes a phosphatase that modulates signaling from upstream receptor tyrosine kinase and the *Ras* genes. In our cohort, we identified only a mutation (G60S) in *PTPN1*1 reported as pathogenic in Noonan syndrome [25] in one patient. Janus kinase family members were also mutated, and novel *JAK1* mutations were found in 3 patients (1 B-ALL and 2 T-ALL), S703I, D604Y and L910P.

#### Mutations in the notch signaling pathway were more common in T-ALL

In our cohort, T-ALL comprised 18.6% (*n* = 26) of ALL patients, and the most commonly mutated genes included *NOTCH1* (23.1%), *FBXW7* (23.1%), *PHF6* (11.5%), *PTEN* (11.5%) and *JAK1* (7.7%). The *Notch* signaling pathway, with the most common abnormality in T-ALL, has important roles in hematopoiesis, angiogenesis, cell proliferation, apoptosis and T cell development. We identified 6 *NOTCH1* mutations, including 4 novel missenses (l1678P, A375G, R1598P and I1616N) and 2 frameshift mutations (Q1455 L fs*25, V2433G fs*35), with the majority of mutations in the heterodimerization domain (HD) (e.g., R1598P, I1616N and L1678P), which led to constitutive activation of the *Notch* pathway (Fig. 2b).

Six unique mutations in *FBXW7*, a component of the E3 ubiquitin ligase complex that controls protein turnover, occurred in 23.1% of T-ALL cases. The well-appreciated activating hotspot mutations R505C (two cases), D399G (one case) in the WD domain, and several novel mutations were identified (Fig. 2b). Notably, two cases (7.7%) included both *NOTCH1* and *FBXW7* mutations, and two cases included both *NOTCH1* and *PHF6* mutations. In addition, a hot spot of the in-frame deletion mutation at codon 6 in *NOTCH2*, another member of the *Notch* family, was observed in 4 cases (3 B-ALL, 1 T-ALL) (Fig. 2b).

#### Alterations in epigenetic regulations

**Fig. 3:**
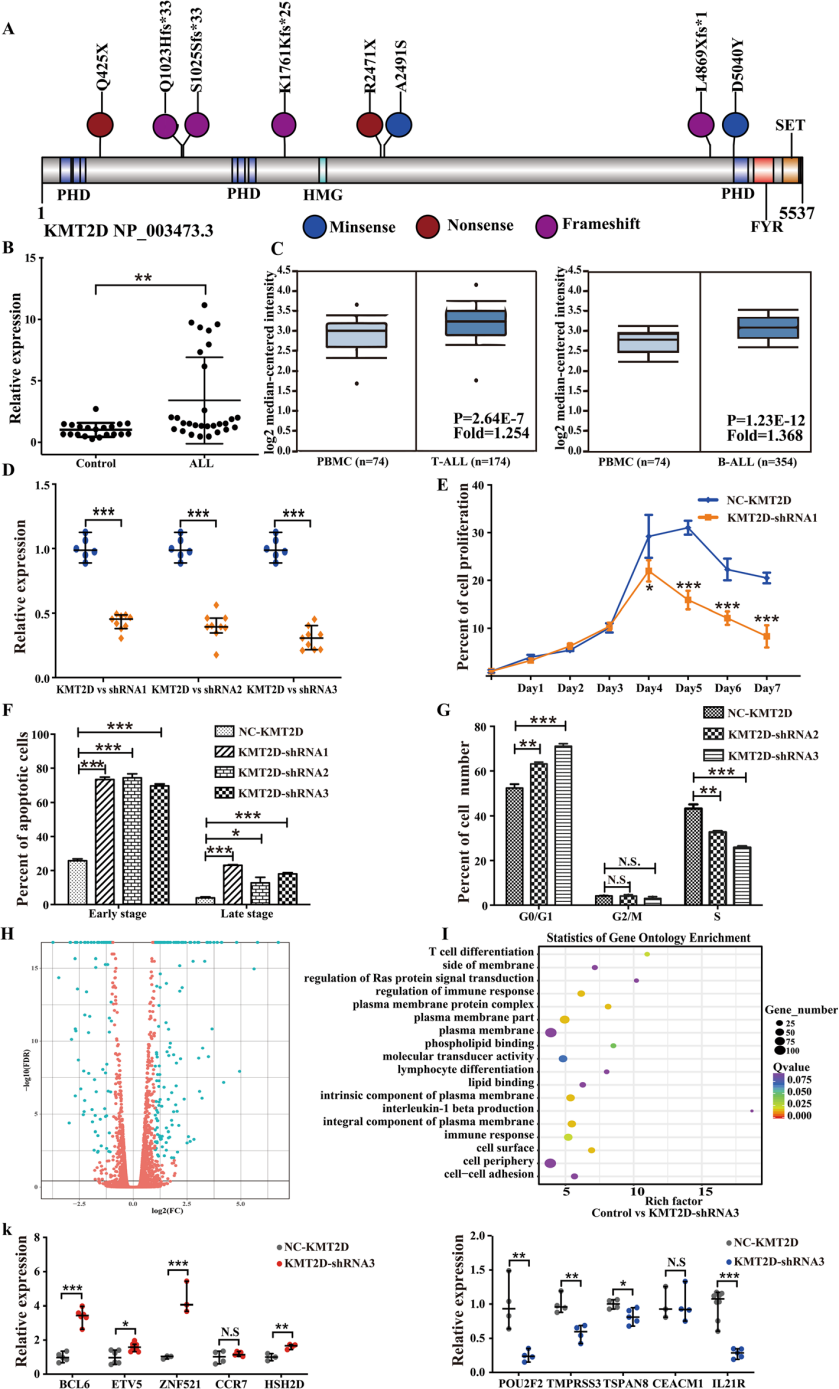
KMT2D is a key oncogene in pediatric ALL. **a** Mutational diagram of *KMT2D*. PHD, plant homeodomain; HMG, high mobility group domain; SET, Su (var)3–9 Enhancer of zeste and Trithorax domain; FYR, FY-rich domain. **b** Increased *KMT2D* mRNA expression in ALL samples. **, *P* < 0.01. **c** Higher expression levels of *KMT2D* in ALL data (retrieved from GSE13159). **d** Generation of Nalm-6 cells with stable knockdown of *KMT2D*. Three shRNA sequences displayed significant suppression of *KMT2D* expression. ***, *P* < 0.001. **e**, **f**, **g** Evaluation of the effect of *KMT2D* knockdown Nalm-6 cells on cell proliferation, cell apoptosis and cell cycle. *, *P* < 0.05; **, *P* < 0.01; ***, *P* < 0.001, N.S., no significance. **h** Volcano plot depicting differentially expressed genes between the *KMT2D* knockdown and control groups. **i** Bubble chart depicts Gene Ontology (GO) functional enrichment analysis of differentially expressed genes. **g** RT-qPCR analysis of selected genes identified as differentially expressed in RNA sequencing. *GAPDH* was used as an endogenous control to normalize for RNA quality. *, *P* < 0.05; **, *P* < 0.01; ***, *P* < 0.001, N.S., no significance

Members of the histone methyltransferase MLL family were mutated frequently. *KMT2A*, known as myeloid/lymphoid or mixed-lineage leukemia (*MLL*), is a well-recognized leukemia-related gene and is rearranged in approximately 75% of infants with B-ALL, particularly in those less than 6 months of age [26]. However, the role of other *MLL* family members in hematological malignancy has not been fully established. In our cohort, we found that *KMT2D*, was the most frequently mutated epigenetic factor. Strikingly, *KMT2D* displayed a higher proportion of inactivating mutations (2 nonsense mutations, 4 frameshift mutations, and 2 missense mutations) (Fig. 3a). This result implied that inactivating mutations lead to a loss of function in a potential tumor suppressor.

However, the function of *KMT2D* in leukemia pathogenesis remains uncharacterized. By examining the gene expression in our patients and related ONCOMINE data (retrieved from GSE13159, the European Leukemia Network), we found that the *KMT2D* gene was highly expressed in both datasets (Fig. 3b, c) [27, 28]. To investigate the functional consequences of the loss-of-function mutations of *KMT2D* in ALL, we stably downregulated the expression of *KMT2D* in Nalm-6 cells using shRNA-mediated gene knockdown approach. We found that all 3 shRNA sequences significantly reduced the expression of *KMT2D* at the mRNA transcript levels (Fig. 3d) and that *KMT2D* knockdown cells exhibited a significant decrease in the cell numbers from day 4 (Fig. 3e). Consistently, downregulation of *KMT2D* promoted the apoptosis of Nalm-6 cells (early stage and late stage, Fig. 3f) and inhibited cell proliferation (significantly increased cell numbers in G0/G1 phase fraction and concomitant decreased in S phase fraction, Fig. 3g). Next, we performed RNA sequencing in both *KMT2D* knockdown and control Nalm-6 cells to examine transcriptomic changes caused by suppression of *KMT2D*. Significantly, 94 genes were upregulated in the *KMT2D* knockdown cells compared with the control cells, whereas 193 genes were downregulated (Fig. 3h). Gene ontology analysis revealed that differentially expressed genes were enriched in immune response, cell plasma membrane and T cell differentiation (Fig. 3i). Using quantitative real-time PCR, we validated the expression changes of several key genes involved in hematopoietic development and immune regulation, including *POU2F2*, *TMPRSS3*, *TSPAN8*, *IL21R* downregulated, and *BCL6*, *ETV5*, *ZNF521*, *HSH2D* upregulated in *KMT2D* knockdown cells (Fig. 3g, Table S3). Together, these findings underscored the critical role of the *KMT2D* gene in lymphoid malignancy and provided a potential therapeutic target for this cancer.

*SETD2* mutations occurred in 2.6% of B-ALL cases and approximately 70% of the *SETD2* lesions were likely to be loss-of-function mutations, including the nonsense mutation (C2525X), frameshift mutations (S165Lfs*12, A158Dfs*13, S1572Xfs*1) and splice site mutations (c.4715(exon5), c.4715 + 1(IVS5) ins TTTTATGAT) (Fig. 2c). Mutations in *CREBBP* occurred in 2 B-ALL patients at 2 new mutational sites (Y1450D, A1473T) (Fig. 2c) in the HAT domain. Inactivating mutations of *PHF6* occurred in 3 T-ALL patients, and 2 cases with *PHF6* mutations co-occurred with *NOTCH1* mutations. However, *EZH2* mutations in the catalytically active SET domain in 2 B-ALL patients, coexisting with *ETV6* mutations (Figure S2). *SUZ12* mutations occurred in only one T-ALL patient, with two types of somatic mutations, and coexisted with *KMT2D* and *TP53* mutations, suggesting a potential interplay of these genes in the pathogenesis.

#### Transcription factors and cell-cycle pathway

Transcription factors *ETV6* and *PAX5* are essential for hematopoietic and lymphoid differentiation. In our cohort, *ETV6* mutations were identified in 4 ALL cases, and *PAX5* mutations were uncovered exclusively in 2 B-ALL cases (Table S5). Other mutated genes are mainly involved in the cell-cycle pathway, including *TP53* and *PTEN*. *TP53* mutations had 4 different types in its DNA-binding domain, including well-known hotspot R273H and other new mutations (Y205D, H179Mfs*68, S166X), these mutations occurred in *TP53* DNA-binding domain may also inactivate *TP53* by affecting its DNA-binding ability [29]. We also found 6 different mutations in *PTEN* (the tumor suppressor phosphatase and tensin homolog) in 3 T-ALL cases (Fig. 2d), as described tumor-associated mutations may occur in all PTEN domains.

In addition, other mutations, including *NBPF10* (*n* = 7), *MDC1* (*n* = 2) and *CCND3* (*n* = 1), were also found in our research (Table S5). The majority of mutations were missense mutations and could be found in other studies, suggesting that these mutations also had significant meaning in ALL.

## Discussion

In this study, we performed a genetic mutational analysis of Chinese children with ALL and identified an abundance of somatic mutations in essential genes, many of which were likely deleterious and may contribute to the pathogenesis of ALL. Although many of the most frequent mutations in pediatric ALL have been described previously, we identified distinct mutational characteristics and influenced different signaling pathways between B-ALL (*Ras* pathway) and T-ALL (*Notch* pathway) in this Chinese cohort. *Ras* pathway mutations were recurrent in pediatric B-ALL [24, 30, 31], and the vast majority of mutations occurred in *KRAS*, *NRAS*, *FLT3* and *NF1*, revealing a central role of these genes in pediatric B-ALL. *Ras* genes mutational sites included G12C/D/S/V, G13D/S/V, Q61K/H, A146T/P and K117N, which were also identified in the study by Ding LW, et al. [32], suggesting that these mutational sites were common in Asian. one patient occurred *KRAS* and *NRAS* mutation simultaneously, these two mutations were close enough to be spanned by the same read-pair allowing the determination if the mutations are on either the same or different alleles [32]. We also found that 75% high-hyperdiploid possessed *FLT3* mutations, which higher than 25% incidence as previous studies [33, 34], indicating a higher incidence in the Chinese patients with ALL associated with hyperdiploidy. Consistent with previous reports [20, 35], we also observed a high incidence of *Ras* pathway mutations in high-hyperdiploid patients with low mutation rates in *TCF3-PBX1* and *MLL* rearrangement cases. Similar research was showed that B-ALL patients carrying any of the recurrent translocations *ETV6-RUNX1*, *BCR-ABL* or *TCF3-PBX1* harbored few mutations compared to the other B-ALL patients [36]. Overall, this further underscores the crucial role of *RAS* mutations in ALL and highlight the genetic heterogeneity of pediatric ALL.

In our cohort, *NOTCH1* mutations occurred in 23.1% of T-ALL cases, which was significantly lower than previously reported values [26, 29]. However, it is interesting that 2 cases with *PHF6* mutations co-occurred with *NOTCH1* mutations and were significantly correlated with the *NOTCH1* mutation in Chinese adult T-ALL (*PHF6 m*ut*NOTCH1m*ut vs *PHF6* wt*NOTCH1m*ut, 75.0% vs 44.2%; *P* = 0.035) [37]. This discrepancy could be caused by the limited number of T-ALL cases enrolled in this study (*n* = 26), or possible coverage bias impairing ability to call gene sequence [38], and the detection of sequence mutations in ALL was insufficient. Frequently, some genes are affected by more than one type of alterations such as point mutation, copy number alterations (CNAs), focal aberrations/small insertions/deletions (INDEL), or structural variations (SVs). So, only one type of analysis lead to the underestimation of the mutation frequency of *NOTCH1*. Similarly, we underestimated the mutation frequencies of *CDKN2A/2B*, *ETV6* and *PAX5*, due to lack of analysis of somatic copy number gains or losses. Copy losses of *CDKN2A/2B* (9p21), *PAX5* (9p13) and *ETV6* (12p13) were prevalent in children, while copy gains of *RUNX1* (21q22.3) were more enriched in children [39]. So, large deletion, amplification and structural variant should be warranted; no single type of sequencing is capable of detecting the same alterations; WES is useful for point mutation investigation, whereas WGS can reveal SVs. Besides, NGS is increasingly being used to monitor drug response and treatment toxicity [40], contributing to the refinement of diagnosis and prognosis for 34% of patients with hematologic malignancies and blood disorders [41]. Incorporating pharmacogenomics and pharmacotranscriptomics can provide an enormous of molecular markers responsible for the efficacy, side effects, and toxicity of the chemotherapeutic drugs to improve the treatment protocols [42]. Then, utilizing genomic technology can better management and potential improve the survival rate in pediatric ALL patients.

In our findings, the most frequently mutated gene of epigenetic regulators was *KMT2D*, which encodes histone methyltransferase for methylates the Lys-4 position of histone H3, and its mutation can cause Kabuki syndrome, an autosomal dominant disease [43]. *KMT2D* is a key regulator of transcriptional enhancer function and plays an important role in maintaining genomic stability [44], and it is mutated in a large number of different cancers (e.g., diffuse large B cell lymphoma, small cell undifferentiated lung cancer, and medulloblastoma) [45–47]. As *KMT2D* is a predicted tumor driver gene in ALL [19] and it overexpressed in ALL, when *KMT2D* is knocked down, it significantly decreased leukemia cell growth, promoted cell apoptosis, and inhibited cell proliferation. A related study also showed that *KMT2D* was overexpressed in primary gastrointestinal diffuse large B cell lymphoma (PGI-DLBCL) and appeared as a prognostic factor for patients older than 60 years old [48]. *KMT2D* overexpression was observed in esophageal squamous cell carcinoma (ESCC), predicting poor clinical outcomes and facilitating ESCC tumor progression [49]. In addition, *KMT2D* can interact with *KMT2A* in acute myeloid leukemia, its deletion reduced *MLL-AF9* leukemia cell survival, and the codeletion of both *KMT2A* and *KMT2D* resulted in more severe reductions in survival, proliferation, and gene expression than either individual gene deletion [50]. Hence, the *KMT2D* gene plays an important role in hematological tumors and may act as a drug target in MLL-rearranged leukemia. However, there existed limitation in our research, the off-target effect remains one of the major obstacles in *KMT2D*-shRNA experiment and it is insufficient to research the function of *KMT2D* in ALL. So, we should generate a *KMT2D* knock-out cells by CRISPR-Cas9-mediated genome editing to demonstrate its potential molecular pathogenesis in ALL in the future study.

As the main part of this study, we intend to show the genomic landscape of pediatric ALL from a single center in China, and our results provided a substantial number of genetic variants contributing to accumulate genetic data of Chinese children and explore molecular determinants in the future. However, there are some limitations in the present study. The number of patients enrolled in the present study was limited, and sample selection may be biased, which may contribute to the discrepancies in the findings between our study and others, and collaborative efforts with larger sample sizes are needed. Structural alterations may play important roles in leukemogenesis; thus, the absence of this information leads to incomplete understanding of the genetic basis of ALL. More comprehensive approaches, such as WGS, RNA-seq, pharmacogenomics and pharmacotranscriptomics, and larger integrative studies, can be warranted to dissect the underlying complexity of ALL in the future. The frequencies and distributions of abnormalities of ALL patients between children and adult, Chinese and western should further be compared in a larger cohort.

## Conclusion

This study provided further insights into the genetic basis of ALL and strengthened that *Ras* mutations were predominant in childhood ALL, especially in the subtype of high-hyperdiploid. These findings have major implications for understanding the genomic complexity of ALL and also have direct implications for the clinical management of ALL.

## Supplementary information

**Additional file 1: Table S1.** 950 Genes screened in the exon sequencing. **Table S2.** Sequences of shRNA used in this study. **Table S3.** Sequences of real-time PCR primers used in this study. **Table S4.** Clinical characteristics and genetic types of patient cohorts. **Table S5.** Other mutations occurring in our ALL cohort. **Figure S1.** Somatic mutations in acute lymphoblastic leukemia (ALL). A, Boxplots showed the median depth of coverage depth in tumor samples and the control samples (matched germline samples). B, Boxplots showed the median number of somatic mutations detected in B-ALL and T-ALL. C, Pattern of single base substitution in B-ALL and T-ALL patients. D, Density plots of the allele fraction (AF) of single nucleotide variants (SNVs) in the B-ALL and T-ALL patients. The main clones with a maximum AF close to 0.4 and subclonal mutations with a maximum AF below 0.25. **Figure S2.** Recurrent mutations in epigenetic regulations. Schematic diagrams of protein structures involving gene mutations in *PHF6, EZH2, SUZ12*.

## Data Availability

The datasets generated and/or analyzed during the current study are available from the corresponding author on reasonable request for privacy reasons.

## Abbreviations

ALL: Acute lymphoblastic leukemia
B-ALL: B cell ALL
FISH: Fluorescence in situ hybridization
HeH: High hyperdiploid
iAMP21: Intrachromosomal amplification of chromosome 21
MAF: Minor allele frequency
MLL: Mixed-lineage leukemia
NGS: Next-generation sequencing
RNA-seq: RNA sequencing
RT-PCR: Reverse transcriptase polymerase chain reaction
shRNAs: Small hairpin RNAs
SNPs: Single-nucleotide polymorphisms
SNV: Single-nucleotide variant
T-ALL: T cell ALL
WBC: White blood cell
WES: Whole exon sequencing
WGS: Whole genome sequencing

## References

[1] Hunger SP, Mullighan CG (2015). Acute lymphoblastic leukemia in children. N Engl J Med.

[2] Wiemels JL, Cazzantiga G, Daniotti M, Eden OB, Addison GM, Masera G (1999). Prenatal origin of acute lymphoblastic leukemia in children. Lancet..

[3] Greaves MF, Wiemels J (2003). Origins of chromosome translocations in childhood leukemia. Nat Rev Cancer.

[4] Holmfeldt L, Wei L, Diaz-Flores E, Walsh M, Zhang J, Ding L (2013). The genomic landscape of hypodiploid acute lymphoblastic leukemia. Nat Genet.

[5] Mullighan CG, Goorha S, Radtke I, Miller CB, Coustan-Smith E, Dalton JD (2007). Genome-wide analysis of genetic alterations in acute lymphoblastic leukemia. Nature..

[6] Inaba H, Geaves M, Mullighan CG (2013). Acute lymphoblastic leukemia. Lancet..

[7] Chiaretti S, Gianfelici V, Ceglie G, Foà R (2014). Genomic characterization of acute leukemia. Med Princ Prac.

[8] Roberts KG, Mullighan CG (2015). Genomics in acute lymphoblastic leukemia: insights and treatment implications. Nat Rev Clin Oncol.

[9] Shah S, Schrader KA, Waanders E, Timms AE, Vijai J, Miething C (2013). A recurrent germline PAX5 mutation confers susceptibility to pre-B cell acute lymphoblastic leukemia. Nat Genet.

[10] Zhang MY, Churpek JE, Keel SB, Walsh T, Lee MK, Loeb KR (2015). Germline ETV6 mutations in familial thrombocytopenia and hematologic malignancy. Nat Genet.

[11] Ma X, Edmonson M, Yergeau D, Muzny DM, Hampton OA, Rusch M (2015). Rise and fall of subclones from diagnosis to relapse in pediatric B-acute lymphoblastic leukemia. Nat Commun.

[12] Mullighan CG, Zhang J, Kasper LH, Lerach S, Payne-Turner D, Phillips LA (2011). CREBBP mutations in relapsed acute lymphoblastic leukemia. Nature..

[13] Malinowska-Ozdowy K, Frech C, Schönegger A, Eckert C, Cazzaniga G, Stanulla M (2015). KRAS and CREBBP mutations: a relapse-linked malicious liaison in childhood high hyperdiploid acute lymphoblastic leukemia. Leukemia..

[14] Tzoneva G, Perezgarcia A, Carpenter Z, Khiabanian H, Tosello V, Allegretta M (2013). Activating mutations in the NT5C2 nucleotidase gene drive chemotherapy resistance in relapsed ALL. Nat Med.

[15] Meyer JA, Wang J, Hogan LE, Yang JJ, Dandekar S, Patel JP (2013). Relapse-specific mutations in NT5C2 in childhood acute lymphoblastic leukemia. Nat Genet.

[16] Li B, Li H, Bai Y, Kirschner-Schwabe R, Yang JJ, Chen Y (2015). Negative feedback-defective PRPS1 mutants drive thiopurine resistance in relapsed childhood ALL. Nat Med.

[17] Roberts KG, Li Y, Payne-Turner D, Harvey RC, Yang YL, Pei D (2014). Targetable kinase-activating Leaions in Ph-like acute lymphoblastic leukemia. N Engl J Med.

[18] Chalandon Y, Thomas X, Havette S, Cayuela JM, Abbal C, Huguet F (2015). Randomized study of reduced-intensity chemotherapy combined with imatinib in adults with Ph-positive acute lymphoblastic leukemia. Blood..

[19] Lindqvist CM, Nordlund J, Ekman D, Johansson A, Moghadam BT, Raine A (2015). The mutational landscape in pediatric acute lymphoblastic leukemia deciphered by whole genome sequencing. Hum Mutat.

[20] Paulsson K, Lilljebjorn H, Biloglav A, Olsson L, Rissler M, Castor A (2015). The genomic landscape of high hyperdiploid childhood acute lymphoblastic leukemia. Nat Genet.

[21] Chen B, Wang Y-Y, Shen Y, Zhang WN, He HY, Zhu YM (2012). Newly diagnosed acute lymphoblastic leukemia in China (I): abnormal genetic patterns in 1346 childhood and adult cases and their comparison with the reports from Western countries. Leukemia..

[22] Fousteri M, Mullenders LH (2008). Transcription-coupled nucleotide excision repair in mammalian cells: molecular mechanisms and biological effects. Cell Res.

[23] Alexandrovi LB, Jones PH, Wedge DC, Sale JE, Campbell PJ, Nik-Zainal S (2015). Clock-like mutational processes in human somatic cells. Nat Genet.

[24] Oshima K, Khiabanian H, da Silva-Almeida AC, Tzoneva G, Abate F, Ambesi-Impiombato A (2016). Mutational landscape, clonal evolution patterns, and role of RAS mutations in relapsed acute lymphoblastic leukemia. Proc Nati Acad Sci US.

[25] Ezquieta B, Santome JL, Carcavilla A, Guillen-Navarro E, Perez-Aytes A (2012). Sanchez del Pozo J, et al. alterations in RAS-MAPA genes in 200 Spanish patients with Noonan and other neuro-cardio-facio-cutaneous syndromes. Genotype and cardiopathy. Rev Esp Cardiol.

[26] Tasian SK, Hunger SP (2017). Genomic characterization of paediatric acute lymphoblastic leukaemia: an opportunity for precision medicine therapeutics. Br J Haematol.

[27] Kohlmann A, Kipps TJ, Rassenti LZ, Downing JR, Shurtleff SA, Mills KI (2008). An international standardization program towards the application of gene expression profiling in routine leukemia diagnostics: the microarray innovations in LEukemia study prophase. Br J Haematol.

[28] Haferlach T, Kohlmann A, Wieczorek L, Basso G, Kronnie GT, Bene MC (2010). Clinical utility of microarray-based gene expression profiling in the diagnosis and subclassification of leukemia: report from the international microarray innovations in leukemia study group. J Clin Oncol.

[29] Liu Y, Easton J, Shao Y, Maciaszek J, Wang Z, Wilkinson MR (2017). The genomic landscape of pediatric and young adult T-lineage acute lymphoblastic leukemia. Nat Genet.

[30] Ariës IM, Van den Dungen RE, Koudijs MJ, Cuppen E, Voest E, Molenaar JJ (2015). Towards personalized therapy in pediatric acute lymphoblastic leukemia: RAS mutations and prednisolone resistance. Haematologica..

[31] Irving J, Matheson E, Minto L, Blair H, Case M, Halsey C (2014). Ras pathway mutations are prevalent in relapsed childhood acute lymphoblastic leukemia and confer sensitivity to MEK inhibition. Blood..

[32] Ding LW, Sun QY, Tan KT, Chien W, Mayakonda A, Yeoh AEJ (2017). Mutational landscape of pediatric acute lymphoblastic leukemia. Cancer Res.

[33] Armstrong SA, Mabon ME, Silverman LB, Li A, Gribben JG, Fox EA (2004). FLT3 mutations in childhood acute lymphoblastic leukemia. Blood..

[34] Braoudaki M, Karpusas M, Katsibardi K, Papathanassiou C, Karamolegou K, Tzortzatou-Stathopoulou F (2009). Frequency of FLT3 mutations in childhood acute lymphoblastic leukemia. Med Oncol.

[35] Jerchel IS, Hoogkamer AQ, Ariës IM, Steeghs EMP, Boer JM, Besselink NJM (2018). RAS pathway mutations as a predictive biomarker for treatment adaptation in pediatric B-cell precursor acute lymphoblastic leukemia. Leukemia..

[36] Lindqvist CM, Lundmark A, Nordlund J, Freyhult E, Ekman D, Almlof JC (2016). Deep targeted sequencing in pediatric acute lymphoblastic leukemia unveils distinct mutational patterns between genetic subtypes and novel relapse-associated genes. Oncotarget..

[37] Li M, Xiao L, Xu J, Zhang R, Guo J, Olson J (2016). Co-existence of PHF6 and NOTCH1 mutations in adult T-cell acute lymphoblastic leukemia. Oncol Lett.

[38] He J, Abdelwahab O, Nahas MK, Wang K, Rampal RK, Intlekofer AM (2016). Integrated genomic DNA/RNA profiling of hematologic malignancies in the clinical setting. Blood..

[39] Liu YF, Wang BY, Zhang WN, Huang JY, Li BS, Zhang M (2016). Genomic profiling of adult and pediatric B-cell acute lymphoblastic leukemia. EBioMedicine..

[40] Heikamp EB, Pui CH (2018). Next-generation evaluation and treatment of pediatric acute lymphoblastic leukemia. J Pediatr.

[41] Marks LJ, Oberg JA, Pendrick D, Sireci AC, Glasser C, Coval C (2017). Precision medicine in children and young adults with hematologic malignancies and blood disorders: the Columbia University experience. Front Pediatr.

[42] Pavlovic S, Kotur N, Stankovic B, Zukic B, Gasic V, Dokmanovic L (2019). Pharmacogenomic and Pharmacotranscriptomic profiling of childhood acute lymphoblastic leukemia: paving the way to personalized treatment. Genes (Basel).

[43] Ng SB, Bigham AW, Buckingham KJ, Hannibal MC, McMillin MJ, Gildersleeve HI (2010). Exome sequencing identifies MLL2 mutations as a cause of kabuki syndrome. Nat Genet.

[44] Kantidakis T, Saponaro M, Mitter R, Horswell S, Kranz A, Boeing S (2016). Mutation of cancer driver MLL2 results in transcription stress and genome instability. Genes Dev.

[45] Pasqualucci L, Dominguez-Sola D, Chiarenza A, Fabbri G, Grunn A, Trifonov V (2011). Inactivating mutations of acetyltransferase genes in B-cell lymphoma. Nature..

[46] Ross JS, Wang K, Elkadi OR, Tarasen A, Foulke L, Sheehan CE (2014). Next-generation sequencing reveals frequent consistent genomic alterations in small cell undifferentiated lung cancer. J Clin Pathol.

[47] Parsons DW, Li M, Zhang X, Jones S, Leary RJ, Lin JC (2011). The genetic landscape of the childhood cancer medulloblastoma. Science..

[48] Ye H, Lu L, Ge B, Gao S, Ma Y, Liang B (2015). MLL2 protein is a prognostic marker for gastrointestinal diffuse large B-cell lymphoma. Int J Clin Exp Pathol.

[49] Abudureheman A, Ainiwaer J, Hou Z, Niyaz M, Turghun A, Hasim A (2018). High MLL2 expression predicts poor prognosis and promotes tumor progression by inducing EMT in esophageal squamous cell carcinoma. J Cancer Res Clin Oncol.

[50] Chen Y, Anastassiadis K, Kranz A, Stewar AF, Arndt K, Waskow C (2017). MLL2, not MLL1, plays a major role in sustaining MLL-rearranged acute myeloid leukemia. Cancer Cell.

