# Supplementary material for: Genetic mutational analysis of pediatric acute lymphoblastic leukemia from a single center in China using exon sequencing
Source: BMC Cancer. 2020 Mar 12;20:211. doi: 10.1186/s12885-020-6709-7 (PMC7068927; doi:10.1186/s12885-020-6709-7)
Supplement: Supplementary file 1 — Additional file 1: Table S1. 950 Genes screened in the exon sequencing. Table S2. Sequences of shRNA used in this study. Table S3. Sequences of real-time PCR primers used in this study. Table S4. Clinical characteristics and genetic types of patient cohorts. Table S5. Other mutations occurring in our ALL cohort. Figure S1. Somatic mutations in acute lymphoblastic leukemia (ALL). A, Boxplots showed the median depth of coverage depth in tumor samples and the control samples (matched germline samples). B, Boxplots showed the median number of somatic mutations detected in B-ALL and T-ALL. C, Pattern of single base substitution in B-ALL and T-ALL patients. D, Density plots of the allele fraction (AF) of single nucleotide variants (SNVs) in the B-ALL and T-ALL patients. The main clones with a maximum AF close to 0.4 and subclonal mutations with a maximum AF below 0.25. Figure S2. Recurrent mutations in epigenetic regulations. Schematic diagrams of protein structures involving gene mutations in PHF6, EZH2, SUZ12. [file 12885_2020_6709_MOESM1_ESM.docx]

Table S1. 950 Genes screened in the exon sequencing

| AARS2 | ARHGAP26 | BCL11B | CBFA2T3 | CHEK1 | CTNNA1 | DNMT3A | ERCC2 |
| --- | --- | --- | --- | --- | --- | --- | --- |
| ABCB1 | ARHGAP32 | BCL2 | CBFB | CHEK2 | CTNNB1 | DOCK2 | ERCC3 |
| ABL1 | ARHGAP35 | BCL2L1 | CBL | CHFR | CTNND1 | DPYD | ERCC4 |
| ABL2 | ARHGAP6 | BCL2L2 | CBLB | CHIC2 | CTTN | DST | ERCC5 |
| ABCC1 | ARHGEF12 | BCL3 | CBLC | CHN1 | CUL1 | DUX4 | ERG |
| ACACA | ARHGEF6 | BCL6 | CCAR1 | CIC | CUX1 | E2F1 | ESR1 |
| ACAT2 | ARID1A | BCL7A | CCDC28A | CIITA | CXCR4 | EBF1 | ESRP1 |
| ACSL3 | ARID1B | BCL9 | CCDC6 | CKS1B | CXCR5 | ECT2L | ETS1 |
| ACSL6 | ARID2 | BCLAF1 | CCM2 | CLCA2 | CXCR7 | EED | ETS2 |
| ACVR1B | ARID5A | BCOR | CCNB1IP1 | CLIP1 | CYLD | EEF1A1 | ETV1 |
| ACVR2A | ARID5B | BCR | CCND1 | CLP1 | CYP1A1 | EEFSEC | ETV4 |
| ADAM10 | ARNT | BIRC2 | CCND2 | CLTC | CYP1B1 | EGF | ETV5 |
| ADAMTS20 | ASGR2 | BIRC3 | CCND3 | CLTCL1 | CYP2C19 | EGFR | ETV6 |
| ADCY1 | ASPM | BIRC5 | CCNE1 | CMC4 | CYP2C8 | EIF3E | EWSR1 |
| ADD3 | ASPSCR1 | BLM | CDH1 | CMPK1 | CYP2D6 | EIF4A2 | EXO1 |
| ADORA2A | ASXL1 | BLNK | CDH11 | CNBP | CYP2E1 | ELF3 | EXT1 |
| AFF1 | ASXL2 | BMPR1A | CDH13 | CNOT3 | DAB2IP | ELF4 | EXT2 |
| AFF3 | ATAD5 | BRAF | CDH2 | CNTRL | DACH1 | ELK4 | EZH2 |
| AFF4 | ATF1 | BRCA1 | CDH20 | COG5 | DAPK1 | ELL | FAM123B |
| AGTRAP | ATG13 | BRCA2 | CDH5 | COL18A1 | DAXX | ELMO1 | FAM175A |
| AHNAK2 | ATIC | BRD1 | CDK12 | COL1A1 | DAZAP1 | ELN | FAM22A |
| AHRR | ATM | BRD3 | CDK2 | COL1A2 | DCLK2 | EMG1 | FAM46C |
| AJUBA | ATP1A1 | BRD4 | CDK4 | COL2A1 | DCTN1 | EML1 | FANCA |
| AKAP6 | ATP2B3 | BRIP1 | CDK5RAP2 | COL6A3 | DDB2 | EML4 | FANCC |
| AKAP9 | ATP5O | BTG1 | CDK6 | COX6C | DDIT3 | EP300 | FANCD2 |
| AKD1 | ATR | BTK | CDK8 | CRBN | DDR2 | EP400 | FANCE |
| AKT1 | ATRX | BUB1B | CDKN1A | CREB1 | DDX10 | EPC1 | FANCG |
| AKT2 | AURKA | CACNA1D | CDKN1B | CREB3L1 | DDX5 | EPCAM | FANCM |
| AKT3 | AURKB | CADM1 | CDKN1C | CREB3L2 | DDX6 | EPHA3 | FAS |
| ALDH2 | AURKC | CALR | CDKN2A | CREBBP | DEK | EPHA7 | FAT1 |
| ALK | AXIN1 | CAMTA1 | CDKN2B | CRKL | DEXI | EPHB1 | FAT2 |
| AMPH | AXIN2 | CANT1 | CDKN2C | CRLF2 | DFFA | EPHB4 | FAT3 |
| ANAPC1 | AXL | CAPRIN1 | CDX2 | CRTC1 | DGKB | EPHB6 | FAT4 |
| ANKRD12 | BAI3 | CARD11 | CEBPA | CRTC3 | DHX9 | EPOR | FBN2 |
| ANKRD28 | BAP1 | CARM1 | CEBPD | CSDE1 | DICER1 | EPS15 | FBXO11 |
| ANTXR1 | BARD1 | CARS | CEP89 | CSF1 | DLG5 | ERBB2 | FBXW7 |
| APAF1 | BAX | CASC5 | CFH | CSF1R | DLX6 | ERBB3 | FCGR2B |
| APC | BAZ2A | CASP10 | CHCHD7 | CSF3R | DMD | ERBB4 | FCGR3A |
| APH1A | BCL10 | CASP8 | CHD4 | CSMD3 | DNM1 | ERC1 | FCRL4 |
| AR | BCL11A | CAST | CHD8 | CTCF | DNM2 | ERCC1 | FDFT1 |

| FES | GFI1 | HMGXB4 | ITGA9 | LEPR | MBD1 | MSI2 | NFATC2 |
| --- | --- | --- | --- | --- | --- | --- | --- |
| FEV | GIT2 | HNF1A | ITGB2 | LHFP | MCL1 | MSN | NFE2L2 |
| FGF9 | GLT6D1 | HNRNPA2B1 | ITGB3 | LIFR | MDC1 | MST1 | NFIB |
| FGFR1 | GMPS | HOXA11 | ITK | LIG1 | MDM2 | MTCP1 | NFKB1 |
| FGFR1OP | GNA11 | HOXA13 | JAK1 | LMNA | MDM4 | MTHFR | NFKB2 |
| FGFR2 | GNAI1 | HOXA9 | JAK2 | LMO1 | MECOM | MTOR | NKAIN2 |
| FGFR3 | GNAQ | HOXB13 | JAK3 | LMO2 | MED12 | MTR | NLRP1 |
| FGFR4 | GNAS | HOXC11 | JARID2 | LNP1 | MED13 | MTRR | NLRP2 |
| FHIT | GOLGA4 | HOXC13 | JAZF1 | LPHN2 | MED17 | MTX1 | NLRP3 |
| FIP1L1 | GOLGA5 | HOXD11 | JUN | LPHN3 | MEF2A | MUC1 | NME1 |
| FLCN | GOPC | HOXD13 | KALRN | LPL | MEF2D | MUC16 | NONO |
| FLI1 | GOT1 | HPRT1 | KAT6A | LPP | MEN1 | MUTYH | NOP2 |
| FLJ27352 | GOT2 | HRAS | KAT6B | LRIG3 | MET | MYB | NOTCH1 |
| FLT3 | GPC3 | HSP90AA1 | KDM5A | LRP1B | METTL14 | MYC | NOTCH2 |
| FLT4 | GPHN | HSP90AB1 | KDM5C | LRRFIP1 | MIPOL1 | MYCL1 | NOTCH4 |
| FN1 | GPR124 | HSPA8 | KDM6A | LRRK2 | MITF | MYCN | NPM1 |
| FNBP1 | GPS2 | HUWE1 | KDSR | LSM14A | MKL1 | MYD88 | NQO1 |
| FOS | GRM8 | ICAM1 | KEAP1 | LZTR1 | MLF1 | MYH11 | NR1H2 |
| FOXA1 | GSK3B | IDH1 | KIAA0284 | MACROD1 | MLH1 | MYH9 | NR4A2 |
| FOXA2 | GSTM1 | IDH2 | KIAA1109 | MAD2L1 | MLH3 | NAB2 | NR4A3 |
| FOXO1 | GSTT1 | IFNG | KIAA1217 | MAGEA1 | MLL | NABP1 | NRAS |
| FOXO3 | GTF2H1 | IGF1R | KIAA1524 | MAGI1 | MLL2 | NARS | NRG1 |
| FOXO4 | GUCY1A2 | IGF2 | KIAA1549 | MALT1 | MLL3 | NAT2 | NSD1 |
| FOXP1 | H3F3A | IGF2R | KIAA1598 | MAML1 | MLLT1 | NAV2 | NT5C2 |
| FOXP4 | H3F3B | IKBKB | KIF1B | MAML2 | MLLT10 | NAV3 | NTRK1 |
| FSTL3 | H3F3C | IKZF1 | KIF5B | MAP2K1 | MLLT11 | NBPF10 | NTRK3 |
| FUBP1 | HAS2 | IL10RB | KIFC3 | MAP2K2 | MLLT3 | NCKIPSD | NUMA1 |
| FUS | HCAR1 | IL2 | KIT | MAP2K4 | MLLT4 | NCOA1 | NUP214 |
| FZD6 | HDAC1 | IL21R | KLF4 | MAP3K1 | MLLT6 | NCOA2 | NUP93 |
| FZR1 | HERPUD1 | IL27 | KLF6 | MAP3K3 | MMP1 | NCOA3 | NUP98 |
| G6PD | HEY1 | IL3 | KLHDC8B | MAP3K4 | MMP2 | NCOA4 | OLIG2 |
| GAB1 | HHEX | IL4 | KLK2 | MAP3K7 | MMP9 | NCOR1 | P2RY8 |
| GAPDH | HIC1 | IL6 | KRAS | MAPK1 | MN1 | NCOR2 | PABPC1 |
| GART | HIF1A | IL6ST | KTN1 | MAPK8 | MNX1 | NCSTN | PAFAH1B2 |
| GAS7 | HIP1 | IL7R | LAMA4 | MAPK8IP1 | MOB2 | NDRG1 | PAK3 |
| GATA1 | HIST1H3B | IL8 | LAMP1 | MAPRE1 | MPL | NEB | PALB2 |
| GATA2 | HIST1H4I | IRF4 | LASP1 | MARK1 | MRE11A | NEDD4L | PARK2 |
| GATA3 | HMGA1 | IRS2 | LAT | MARK4 | MRPS10 | NEFH | PARP1 |
| GATA5 | HMGA2 | ITGA10 | LATS2 | MAST2 | MSH2 | NF1 | PATZ1 |
| GDNF | HMGB3 | ITGA6 | LEF1 | MATK | MSH6 | NF2 | PAX3 |

| PAX5 | POU5F1 | RAD54L | SDHA | SOX17 | TCF12 | TOP2A | WWOX |
| --- | --- | --- | --- | --- | --- | --- | --- |
| PAX7 | POU6F2 | RAF1 | SDHB | SOX2 | TCF3 | TOP3A | WWTR1 |
| PAX8 | PPARG | RALGDS | SDHC | SPATA6 | TCF7 | TP53 | XPA |
| PBRM1 | PPFIBP1 | RANBP17 | SDHD | SPECC1 | TCF7L2 | TP53BP1 | XPC |
| PBX1 | PPM1D | RANBP2 | SEC31A | SPEN | TCL1A | TP63 | XPO1 |
| PCBP1 | PPP2R1A | RANBP3 | SENP6 | SPG20 | TERT | TP73 | XRCC1 |
| PCM1 | PPP2R2A | RAP1GDS1 | SEPT11 | SPOP | TET1 | TPM3 | XRCC2 |
| PCSK7 | PRDM1 | RARA | SEPT2 | SPRTN | TET2 | TPM4 | XRCC3 |
| PDCD10 | PRDM16 | RASA1 | SEPT5 | SPRY3 | TFAP2B | TPMT | XRCC4 |
| PDCD1LG2 | PREB | RASSF1 | SEPT6 | SQSTM1 | TFDP1 | TPR | YTHDF2 |
| PDE4DIP | PRF1 | RB1 | SEPT7 | SRGAP3 | TFE3 | TRAF7 | YWHAE |
| PDGFRA | PRKAA1 | RBM15 | SEPT9 | SRSF2 | TFEB | TRIM24 | ZBTB16 |
| PDGFRB | PRKAR1A | RBM6 | SET | SRSF3 | TFG | TRIM27 | ZBTB5 |
| PER1 | PRKCD | RBMX | SETBP1 | SS18L1 | TFPT | TRIM33 | ZCCHC8 |
| PEX5 | PRKDC | RECQL4 | SETD2 | SSX1 | TFRC | TRIM7 | ZFHX3 |
| PGAP3 | PRKG2 | RELA | SF3B1 | SSX2 | TGFBR1 | TRIO | ZFP36L1 |
| PHF1 | PRPF8 | RELN | SH2B3 | SSX4 | TGFBR2 | TRIP11 | ZFP36L2 |
| PHF6 | PRRX1 | RET | SH2D1A | STAG1 | TGIF1 | TRRAP | ZFYVE19 |
| PICALM | PRRX2 | RMI2 | SH3GL1 | STAG2 | TGM6 | TSC1 | ZMIZ1 |
| PIK3C2B | PSEN1 | RNASEL | SLC1A2 | STAT1 | TGM7 | TSC2 | ZMYM2 |
| PIK3CA | PSMA4 | RNF115 | SLC22A18 | STAT3 | THBS1 | TSG101 | ZNF132 |
| PIK3CB | PTCH1 | RNF2 | SLC34A2 | STAT5B | THRAP3 | TSHR | ZNF300 |
| PIK3CD | PTEN | RNF213 | SLC45A3 | STAT6 | TIAM1 | TTL | ZNF331 |
| PIK3CG | PTGS2 | RNF43 | SLC52A3 | STIL | TIMP1 | TUBA1A | ZNF384 |
| PIK3R1 | PTPN1 | ROS1 | SLCO1B3 | STK11 | TIMP3 | TXNIP | ZNF521 |
| PIK3R2 | PTPN11 | RPGR | SMAD2 | STK36 | TIMP4 | U2AF1 | ZNF814 |
| PIP5K1A | PTPN2 | RPL10 | SMAD3 | STK4 | TLR4 | UBR5 | ZNF844 |
| PKHD1 | PTPN3 | RPL22 | SMAD4 | SUZ12 | TLX1 | UGT1A1 | ZNF91 |
| PKLR | PTPRB | RPL5 | SMAD7 | SYNE1 | TLX3 | USP42 | ZRSR2 |
| PLAG1 | PTPRC | RPN1 | SMARCA4 | SYNPO2 | TMPRSS2 | USP6 |  |
| PLCE1 | PTPRD | RPS6KA2 | SMARCB1 | TAF1 | TNFAIP3 | USP9X |  |
| PLCG1 | PTPRK | RPTN | SMARCD1 | TAF15 | TNFRSF10C | VEGFA |  |
| PLEC | PTPRT | RRM1 | SMARCE1 | TAF1L | TNFRSF14 | VTI1A |  |
| PLEKHG5 | RAD21 | RSPO2 | SMC1A | TAL1 | TNFRSF17 | WAS |  |
| PML | RAD50 | RSPO3 | SMUG1 | TAL2 | TNFRSF19 | WDR48 |  |
| PMS1 | RAD51 | RUNX1 | SNX2 | TBL1XR1 | TNFRSF8 | WHSC1 |  |
| PMS2 | RAD51B | RUNX1T1 | SNX29 | TBX22 | TNK1 | WHSC1L1 |  |
| POT1 | RAD51C | SACM1L | SOCS1 | TBX3 | TNK2 | WIF1 |  |
| POTEF | RAD51D | SAMD9 | SOD2 | TBXAS1 | TOM1 | WRN |  |
| POU2AF1 | RAD54B | SDC4 | SOX11 | TCEA1 | TOP1 | WT1 |  |

Table S2. Sequences of shRNA used in this study

| shRNA | Sequence |
| --- | --- |
| KMT2D-shRNA1 | CCTAGCAGAAACCCAGAAG CTTCCTGTCAGACTTCTGGGTTTCTGCTAGG |
| KMT2D-shRNA2 | GCAGTTTGTGCACTCCAAGCTTCCTGTCAGACTTGGAGTGCACAAACTGC |
| KMT2D-shRNA3 | CCTGAATTGAACAACAGTCTTCTTCCTGTCAGAAAGACTGTTGTTCAATTCAGG |

| GAPDH | F- GGAGCGAGATCCCTCCAAAAT |
| --- | --- |
|  | R- GGCTGTTGTCATACTTCTCATGG |
| KMT2D | F-GGAATGGGTAGCTCTTTGGCGA |
|  | R- TGCCGAATCAGCAGCTCTCGTA |
| BCL6 | F-GGAGTCGAGACATCTTGACTGA |
|  | R-ATGAGGACCGTTTTATGGGCT |
| ETV5 | R-TGCCGGTCTTCCCGAAATG |
|  | F-TCAGCAAGTCCCTTTTATGGTC |
| CEACAM1 | F-TGCTCTGATAGCAGTAGCCCT |
|  | R-GCTCTTCAGAATCGTGAGCCA |
| ZNF521 | F-AAGCAAGCGAAACCGAGATCC |
|  | R-GCCTCTTCTTACAATCTAGTGCC |
| IL-21R | F-GGCAAGACCAGTATGAAGAGC |
|  | R-TGACACTGAAAATGTCGTCGG |
| HSH2D | F-TGTGAACTTGTCGTCACTCTTG |
|  | R-GAGGGGCTTTTGAGAGATGTG |
| CCR7 | F-TGAGGTCACGGACGATTACAT |
|  | R-GTAGGCCCACGAAACAAATGAT |
| POU2F2 | F-GAGGAGCCCAGTGATCTGGA |
|  | R-GAAGCGGGAAATGGTCGTC |
| TMPRSS3 | F-TGGAAGGGTCACTACGCAAAT |
|  | R-AGTGGTGTAATGCAGTCACCT |
| TSPAN8 | F-GGATGCTGCGGTGCTATAAAA |
|  | R-TTCATTCACAATGCGATCAGACT |

Table S3. Sequences of real-time PCR primers used in this study

Table S4. Clinical characteristics and genetic types of patient cohorts

|  | No. of patients (%) |
| --- | --- |
| Total | 140 |
| Immunophenotyped |  |
| B-ALL | 114 (81.4%) |
| T-ALL | 26 (18.6%) |
| Age at diagnosis (years) |  |
| Median | 5.37 |
| Range | 0.3-13.8 |
| Gender |  |
| Male | 86 (61.4%) |
| Female | 54 (38.6%) |
| WBC count at diagnosis (× 10^9^/L) |  |
| Median | 5.55 |
| Range | 0.5-445.0 |
| Genetic abnormalities |  |
| *ETV6-RUNX1* | 31 |
| HeH | 30 |
| *BCR-ABL1* | 9 |
| *SIL-TAL1* | 5 |
| *MLLr* | 4 |
| *TCF3-PBX1* | 3 |
| *iAMP21* | 2 |

Abbreviation: B-ALL, B cell ALL; T-ALL, T cell ALL; HeH, high-hyperdiploid (51~67 chromosomes); iAMP21, Intrachromosomal amplification of chromosome 21.

Table S5. Other mutations occurring in our ALL cohort

| Gene | Location | Nucleotide change | Protein change | rs |
| --- | --- | --- | --- | --- |
| AHNAK2 | 14q32.33 | c.3407(exon7)C>G | p.P1136R | rs201832586 |
|  |  | c.7948(exon7)delA | p.M2650Cfs*27 |  |
| BCL11B | 14q32.2 | c.1415(exon4)G>A | p.R472H |  |
| BCOR | Xp11.4 | c.4675(exon12)G>C | p.A1559P |  |
| CCND3 | 6p21 | c.626(exon5)T>C | p.I209T |  |
| CHD4 | 12p13 | c.2197(exon15)G>C | p.G733R |  |
| CHD8 | 14q11.2 | c.971(exon2)T>A | p.L324Q |  |
| CNOT3 | 19q13.4 | c.724(exon9)_c.725(exon9)insC | p.S242Sfs*11 |  |
| CTCF | 16q21-q22.3 | c.1024(exon5)C>T | p.R342C |  |
|  |  | c.1309(exon7)_c.1310(exon7)delTT | p.F437Sfs*6 |  |
| DUX4 | 4q35 | c.43(exon1)_c.44(exon1)insGGCT | p.R15Rfs*25 |  |
|  |  | c.77(exon1)T>C | p.L26P |  |
|  |  | c.105(exon1)T>G | p.S35R |  |
| EP300 | 22q13.2 | c.5414(exon31)_c.5415(exon31)  insATGATA | p.F1805delinsLX,478 |  |
| EPOR | 19p13.3-p13.2 | c.1220(exon8)C>G | p.S407X,102 |  |
| ERBB2 | 17q12 | c.1669(exon18)T>C | p.C557R |  |
| ERBB3 | 12q13 | c.3380(exon27)G>A | p.R1127H | rs2271188 |
| ERG | 21q22.3 | c.1200(exon12)_c.1201(exon12)insCGGT | p.G401Rfs*73 |  |
|  |  | c.1129(exon12)C>T | p.R377C |  |
| ETV6 | 12p13 | c.163+1(IVS2)G>A |  |  |
|  |  | c.641(exon5)C>T | p.P214L | rs724159947 |
|  |  | c.722(exon5)_c.723(exon5)insACCC | p.E241Efs*4 |  |
| GATA3 | 10p15 | c.826(exon4)C>T | p.R276W |  |
| GATA5 | 20q13.33 | c.874(exon5)C>T | p.R292W |  |
| JAK1 | 1p32.3-p31.3 | c.2108(exon15)G>T | p.S703I |  |
|  |  | c.1810(exon13)G>T | p.D604Y |  |
|  |  | c.2729(exon20)T>C | p.L910P |  |
| JAK3 | 19p13.1 | c.2635(exon19)C>G | p.H879D |  |
| JARID2 | 6p24-p23 | c.1265(exon7)T>G | p.V422G | rs867980083 |
| KDM5A | 12p11 | c.2372(exon17)C>T | p.A791V | rs199735103 |
| KDM6A | Xp11.2 | c.475(exon6)_c.476(exon6)insC | p.V159Afs*2 |  |
| LRP1B | 2q21.2 | c.13739(exon91)A>G | p.D4580G |  |
|  |  | c.3497(exon22)G>C | p.G1166A |  |
| MAP3K3 | 17q23.3 | c.1361(exon14)G>A | p.R454H |  |
| MDC1 | 6p21.3 | c.4831(exon10)C>A | p.P1611T |  |
|  |  | c.4831(exon10)C>A | p.P1611T |  |
|  |  | c.4316(exon10)G>T | p.R1439M |  |
|  |  | c.3551(exon10)C>A | p.S1184Y |  |
|  |  | c.4831(exon10)C>A | p.P1611T |  |
| MLL | 11q23 | c.3568(exon5)_c.3569(exon5)delAA | p.K1190Dfs*21 |  |
|  |  | c.3569+2(IVS5)_c.3569+20(IVS5)  delTAAGTGGGTGTTTCACTCT |  |  |
| MLL3 | 7q36.1 | c.3340(exon21)T>C | p.C1114R | rs200559566 |
|  |  | c.2591(exon15)A>G | p.E864G | rs4024420 |
|  |  | c.1013(exon8)C>T | p.S338L | rs763762478 |
|  |  | c.1013-2(IVS7)A>G |  | rs751158858 |
|  |  | c.1042(exon8)G>A | p.D348N | rs201834857 |
|  |  | c.3401(exon21)G>A | p.S1134N | rs4024339 |
| NARS | 18q21.31 | c.1403(exon13)A>G | p.N468S | rs182629969 |
| NBPF10 | 1q21.1 | c.580(exon5)G>A | p.A194T | rs12124464 |
|  |  | c.5157(exon40)C>A | p.N1719K | rs372069112 |
|  |  | c.8410(exon67)C>A | p.Q2804K | rs200523906 |
| NCOR1 | 17p11.2 | c.1048(exon10)C>T | p.R350X,2091 |  |
|  |  | c.919(exon10)_c.920(exon10)insA | p.I307Nfs*6 |  |
|  |  | c.551(exon5)G>T | p.S184I |  |
| NCOR2 | 12q24 | c.3755(exon30)C>T | p.P1252L | rs371001060 |
| NF1 | 17q11.2 | c.3916(exon29)C>T | p.R1306X,1534 |  |
|  |  | c.1954(exon17)delC | p.R652Vfs*36 |  |
| NSD1 | 5q35 | c.5932(exon19)G>A | p.E1978K |  |
|  |  | c.2608(exon5)A>G | p.R870G |  |
| NT5C2 | 10q24.32 | c.1366(exon18)C>T | p.R456C |  |
| PAX5 | 9p13 | c.88(exon2)G>A | p.G30R |  |
|  |  | c.166(exon2)A>G | p.R56G |  |
| PER1 | 17p13.1 | c.388(exon4)G>T | p.A130S |  |
|  |  | c.460(exon4)C>T | p.R154W | rs147306825 |
| PLEC | 8q24 | c.13904(exon32)G>A | p.R4635Q | rs782435339 |
|  |  | c.100(exon1)C>T | p.R34C | rs782758199 |
| PIK3C2B | 1q32 | c.932(exon3)C>T | p.P311L | rs200476927 |
| PIK3R1 | 5q13.1 | c.1737(exon13)_c.1738(exon13)delAT | p.Q579Qfs*22 |  |
| PRPF8 | 17p13.3 | c.2504(exon17)A>G | p.D835G |  |
|  |  | c.6245(exon39)A>G | p.N2082S | rs546321997 |
| PTPN11 | 12q24 | c.178(exon3)G>A | p.G60S | rs397507507 |
| PTPRT | 20q12-q13 | c.1115(exon7)C>A | p.P372Q |  |
|  |  | c.4278(exon31)C>G | p.F1426L | rs77576329 |
| RECQL4 | 8q24.3 | c.1687(exon10)G>A | p.E563K |  |
|  |  | c.2500(exon15)G>A | p.A834T | rs767722327 |
| RUNX1T1 | 8q22 | c.866(exon10)G>A | p.R289Q | rs866302796 |
| SYNE1 | 6q25 | c.11386(exon70)C>A | p.Q3796K |  |
|  |  | c.7786(exon52)C>T | p.R2596C | rs768574390 |
| TBL1XR1 | 3q26.32 | c.1108(exon12)G>A | p.D370N | rs1057517933 |
| TRRAP | 7q21.2-q22.1 | c.9406(exon62)G>A | p.E3136K |  |


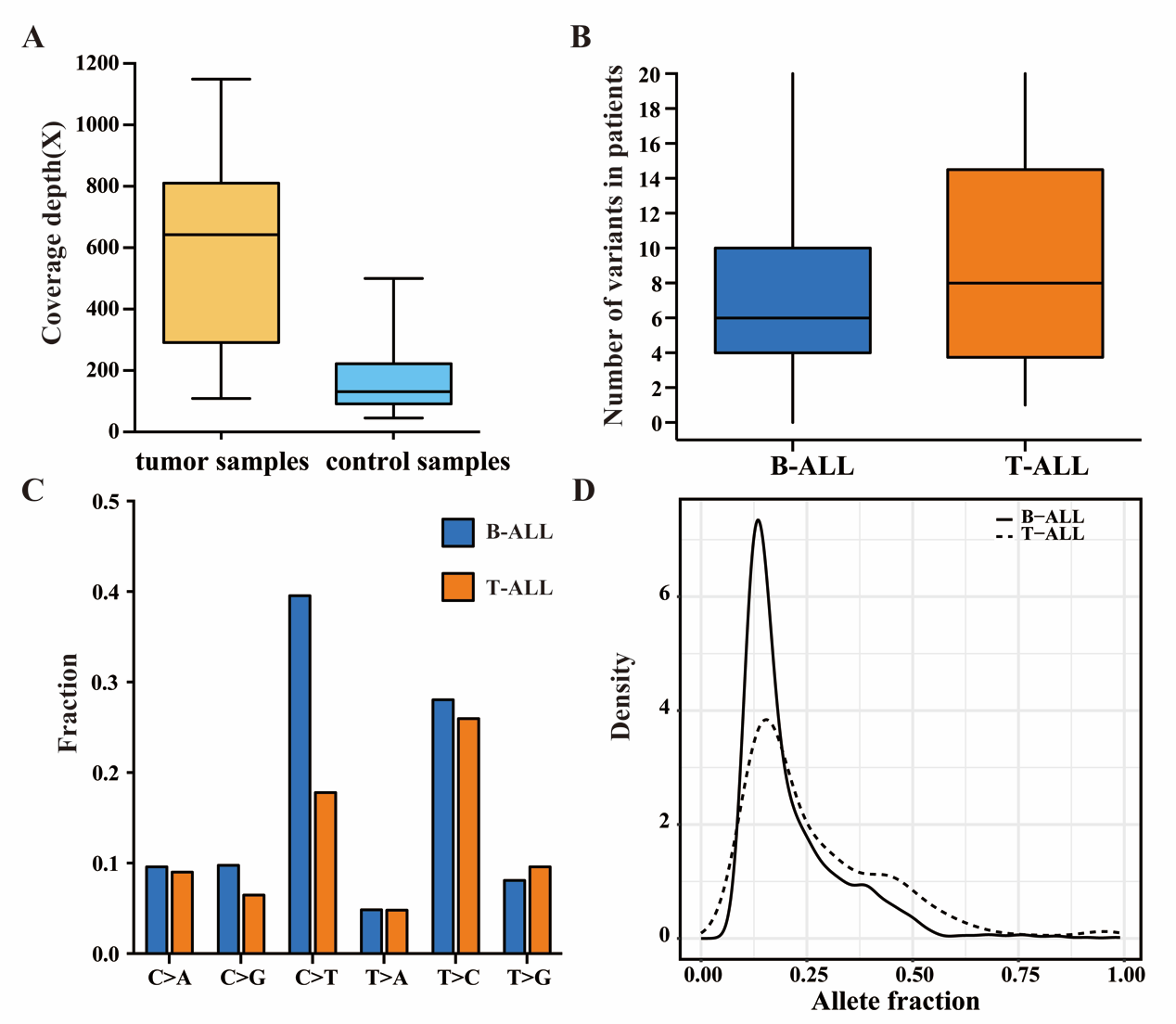
Figure S1. Somatic mutations in acute lymphoblastic leukemia (ALL)

A, Boxplots showed the median depth of coverage depth in tumor samples and the control samples (matched germline samples).

B, Boxplots showed the median number of somatic mutations detected in B-ALL and T-ALL.

C, Pattern of single base substitution in B-ALL and T-ALL patients;

D, Density plots of the allele fraction (AF) of single nucleotide variants (SNVs) in the B-ALL and T-ALL patients. The main clones with a maximum AF close to 0.4 and subclonal mutations with a maximum AF below 0.25.


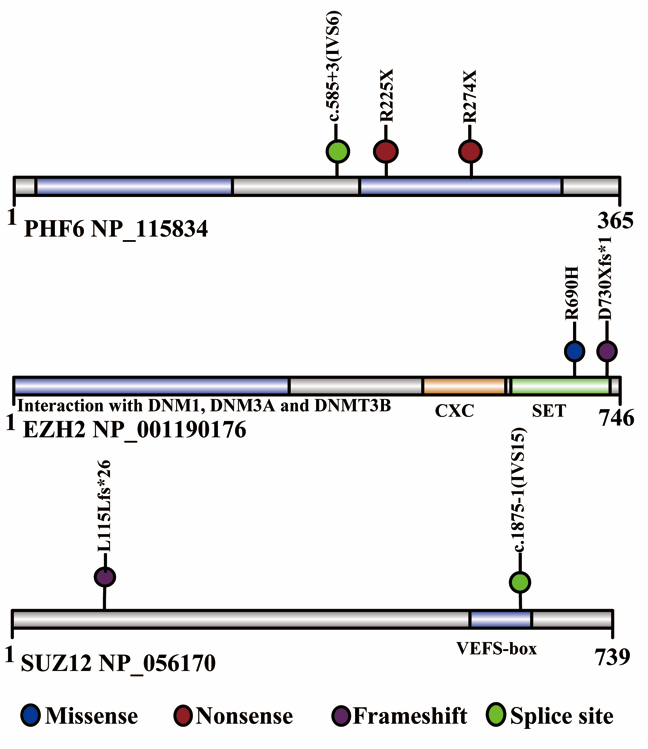


Figure S2. Recurrent mutations in epigenetic regulations

Schematic diagrams of protein structures involving gene mutations in PHF6, EZH2, SUZ12.
